# Genome Sequence of a Complex HIV-1 Unique Recombinant Form Involving CRF01_AE, Subtype B, and Subtype B′ Identified in Malaysia

**DOI:** 10.1128/genomeA.00139-14

**Published:** 2014-03-27

**Authors:** Wei Zhen Chow, Sarimie Nizam, Lai Yee Ong, Kim Tien Ng, Kok Gan Chan, Yutaka Takebe, Clayton Koh, Adeeba Kamarulzaman, Kok Keng Tee

**Affiliations:** a Centre of Excellence for Research in AIDS (CERiA), Department of Medicine, Faculty of Medicine, University of Malaya, Kuala Lumpur, Malaysia; b Division of Genetics and Molecular Biology, Institute of Biological Sciences, Faculty of Science, University of Malaya, Kuala Lumpur, Malaysia; c AIDS Research Center, National Institute of Infectious Diseases, Toyama, Shinjuku-ku, Tokyo, Japan

## Abstract

A complex HIV-1 unique recombinant form involving subtypes CRF01_AE, B, and B′ was recently identified from an injecting drug user in Malaysia. A total of 13 recombination breakpoints were mapped across the near-full-length genome of isolate 10MYPR226, indicating the increasingly diverse molecular epidemiology and frequent linkage among various high-risk groups.

## GENOME ANNOUNCEMENT

Human immunodeficiency virus type 1 (HIV-1) is characterized by extensive genetic diversity due to the error-prone reverse transcriptase, high viral turnover rates, and genetic recombination (1). HIV-1 group M consists of nine subtypes (A to D, F to H, J, and K) and recently, 61 circulating recombinant forms (CRFs) (http://www.hiv.lanl.gov). In Asia, the wide cocirculation and dual infection of CRF01_AE and subtypes B of western origin and B′ of Thai origin that were primarily associated with the epidemics among heterosexuals, men who have sex with men (MSM), and people who inject drugs, respectively, may play an important role in the emergence of diverse intergenotype recombinants. Most CRFs reported from Southeast Asia to date are composed of CRF01_AE and subtype B′: CRF15_01B and CRF34_01B in Thailand, CRF33_01B, CRF48_01B, CRF53_01B, CRF54_01B, and CRF58_01B in Malaysia, and CRF52_01B both in Thailand and Malaysia (2–9). Newly identified CRFs among MSM were composed of CRF01_AE and subtype B: CRF51_01B in Singapore, and CRF55_01B and CRF59_01B in China (10–12). In this study, we characterized the near-full-length genome of a complex HIV-1 unique recombinant form (URF), HIV isolate 10MYPR226, from an injecting drug user in Malaysia.

The near-full-length genome was sequenced from a blood plasma sample collected from a 53-year-old male subject in September 2010. At the sampling time, the CD4 cell count and HIV-1 RNA viral load measurements were 182 cells/µl and 50,141 copies/ml, respectively. HIV-1 RNA was extracted by magnetic bead-based purification method using NucliSENS easyMAG (bioMérieux, France) and reverse transcribed using SuperScript III RT (Invitrogen, USA). The near-full-length genome was amplified by nested PCR using HotStar Taq *Plus* DNA polymerase (Qiagen, USA) to produce 10 overlapping fragments using primers, sequenced with ABI Prism 3730xl (Applied Biosystems, USA), and assembled prior to alignment, phylogenetic, and recombination analyses (similarity plot, bootscan, and informative sites estimation) (8).

The near-full-length genome of 10MYPR226 is 9,007 bp in size (HXB2 nucleotide [nt] positions 642 to 9633) and spanned the *gag*, *pol*, *env*, *tat*, *rev*, *vif*, *vpr*, *vpu*, and *nef* genes. Recombination detection analyses using CRF01_90THCM240, B_83HXB2, and B′_93CNRL42 as putative parental subtypes revealed a complex intersubtype recombination between CRF01_AE, subtype B, and subtype B′ across the genome. Seven CRF01_AE segments were identified at the following HXB2 nucleotide positions: 1197 to 1986 (790 bp), 3194 to 3405 (212 bp), 3788 to 4241 (454 bp), 4463 to 4632 (170 bp), 4988 to 6369 (1382 bp), 8300 to 8504 (205 bp), and 9009 to 9633 (625 bp). Two subtype B segments were present in the *pol* gene at HXB2 nucleotide positions 2064 to 3189 (1126 bp) and 4253 to 4445 (193 bp). Five other subtype B′ segments were identified at HXB2 nucleotide positions 642 to 1168 (527 bp), 3422 to 3734 (313 bp), 4637 to 4775 (139 bp), 6410 to 8244 (1835 bp), and 8547 to 8957 (411 bp). A total of 13 recombination breakpoints were estimated between these regions. Subregion neighbor-joining phylogenetic reconstructions performed using established HIV-1 reference sequences confirmed the putative parental origins of each recombinant segment. To our knowledge, this is the first report of a complex HIV-1 URF descended from three major genetic lineages circulating in the region, indicating frequent linkage among various high-risk groups.

### Nucleotide sequence accession number.

The genome sequence of 10MYPR226 has been deposited in GenBank under the accession no. KJ206289.
